# Dipeptidyl peptidase IV inhibitor attenuates kidney injury in rat remnant kidney

**DOI:** 10.1186/1471-2369-14-98

**Published:** 2013-04-27

**Authors:** Kwon Wook Joo, Sejoong Kim, Shin-young Ahn, Ho Jun Chin, Dong-Wan Chae, Jeonghwan Lee, Jin Suk Han, Ki Young Na

**Affiliations:** 1 Department of Internal Medicine, Seoul National University Hospital, Seoul, South Korea; 2 Department of Internal Medicine, Seoul National University College of Medicine, Seoul, South Korea; 3 Department of Internal Medicine, Seoul National University Bundang Hospital, 166 Gumi-ro, Bundang-gu, Seongnam-si, Gyeonggi-do, 463-707, South Korea

**Keywords:** Dipeptidyl peptidase IV, Glucagon-like peptide-1 receptor, FoxO3a, Sitagliptin, Kidney injury

## Abstract

**Background:**

The inhibition of dipeptidyl peptidase (DPP) IV shows protective effects on tissue injury of the heart, lung, and kidney. Forkhead box O (FoxO) transcriptional factors regulate cellular differentiation, growth, survival, the cell cycle, metabolism, and oxidative stress. The aims of this study were to investigate whether the DPP IV inhibitor sitagliptin could attenuate kidney injury and to evaluate the status of FoxO3a signaling in the rat remnant kidney model.

**Methods:**

Rats were received two-step surgery of 5/6 renal mass reduction and fed on an oral dose of 200 mg/kg/day sitagliptin for 8 weeks. Before and after the administration of sitagliptin, physiologic parameters were measured. After 8 weeks of treatment, the kidneys were harvested.

**Results:**

The sitagliptin treatment attenuated renal dysfunction. A histological evaluation revealed that glomerulosclerosis and tubulointerstitial injury were significantly decreased by sitagliptin. Sitagliptin decreased DPP IV activity and increased the renal expression of glucagon-like peptide-1 receptor (GLP-1R). The subtotal nephrectomy led to the activation of phosphatidylinositol 3-kinase (PI3K)-Akt and FoxO3a phosphorylation, whereas sitagliptin treatment reversed these changes, resulting in PI3K-Akt pathway inactivation and FoxO3a dephosphorylation. The renal expression of catalase was increased and the phosphorylation of c-Jun N-terminal kinase (JNK) was decreased by sitagliptin. Sitagliptin treatment reduced apoptosis by decreasing cleaved caspase-3 and −9 and Bax levels and decreased macrophage infiltration.

**Conclusions:**

In rat remnant kidneys, DPP IV inhibitor attenuated renal dysfunction and structural damage. A reduction of apoptosis, inflammation and an increase of antioxidant could be suggested as a renoprotective mechanism together with the activation of FoxO3a signaling. Therefore, DPP IV inhibitors might provide a promising approach for treating CKD, but their application in clinical practice remains to be investigated.

## Background

Glucagon-like peptide-1 (GLP-1) is a gut incretin hormone, whose mimetics have been used as a therapeutic agent for type 2 diabetes. It stimulates pancreatic beta cell proliferation and insulin secretion in a glucose-dependent manner [1]. However, this peptide is almost immediately degraded by dipeptidyl peptidase (DPP) IV in the circulation. DPP IV has a wide variety of substrates that have important roles in cell migration and differentiation, glucose regulation, metabolism, and inflammation [2]. Sitagliptin, a highly selective DPP IV inhibitor, is currently used in the treatment of type 2 diabetes patients to improve glucose tolerance by increasing the half-life of GLP-1 and glucose-dependent insulinotropic peptide (GIP) [3].

The GLP-1 receptor (GLP-1R) agonist exendin-4 has been reported to ameliorate diabetic nephropathy in animals [4,5]. Recently, studies have shown that DPP IV inhibitors attenuate kidney injury in diabetic animal models [6,7]. In addition to diabetic nephropathy, DPP IV inhibition protected the kidney against ischemia-reperfusion injury (IRI) [8]. Tissue protective effects of GLP-1 activation or DPP IV inhibition have also been demonstrated in other organs, including IRI of the lung during transplantation [9-11] and the outcome of myocardial infarction [12,13].

Most cases of chronic kidney disease (CKD) inevitably progress to end-stage renal disease, which has a high associated morbidity and mortality. Although the initiating insult of CKD is variable, the progression of the disease seems to be common to all kidney diseases that involve a vicious cycle of nephron destruction, glomerulosclerosis and tubulointerstitial fibrosis [14]. However, few pharmacologic treatments have been shown to attenuate the progression of CKD. The remnant kidney is a disease model that mimics the progression of CKD in humans. In this model, there is early glomerulosclerosis by week 4, with segmental sclerosis with tubulointerstitial fibrosis by week 8. Animals die of uremia starting at week 12 to week 16 [15]. Therefore, 8 weeks after subtotal nephrectomy must be a proper time to observe renal pathology in this model.

Forkhead box O (FoxO) transcriptional factors regulate various downstream target genes, including those involved in cellular differentiation, growth, survival, the cell cycle, glucose and lipid metabolism, stress, and the detoxification of reactive oxygen species (ROS) [16]. The phosphatidylinositol 3-kinase (PI3K) and serine–threonine kinase Akt/PKB (Akt) pathway regulates FoxO through phosphorylation. The Akt-mediated phosphorylation of FoxO inhibits the activity of FoxO by promoting its interaction with 14-3-3 proteins and its nuclear exportation, and also by inducing its degradation by the proteasome [17]. In the kidney, the FoxO3 transcript is the most abundant among four subfamily members of FoxO proteins [18]. However, there is no study on the status of FoxOs in the remnant kidney model.

Based on the previous reports of tissue protective effects, we hypothesize that DPP IV inhibition could have a positive effect on this animal model of CKD. Due to the diverse regulatory functions of FoxO, our hypothesis is that its signaling might also be modulated by DPP IV inhibition in this model. Therefore, it would be interesting to investigate FoxO signaling in the kidneys. The present study aimed to evaluate whether sitagliptin could attenuate kidney injury in a rat remnant kidney model. Moreover, we investigated the status of FoxO3a signaling after sitagliptin treatment in this model. To do this, we made CKD animal model by two-step surgery of 5/6 renal mass reduction, and then fed these rats on a 200 mg/kg/day of sitagliptin for 8 weeks.

## Methods

### Animal experiments

All animal procedures were approved by the Institutional Animal Care and Use Committee of the Medical Science Research Institute, Seoul National University Bundang Hospital (BA 1112-095/080-01). Male Sprague–Dawley rats (Orient Bio Inc., Seongnam, South Korea) weighing approximately 200 g were used. The rats (*n* = 21) were randomly assigned to three groups: sham-operation (sham), subtotal nephrectomy (Nx), and subtotal nephrectomy + sitagliptin treatment (Nx+STG) groups. After a right subcostal incision, the right kidney was exposed and separated from the adrenal gland under anesthesia with enflurane (Choongwae Pharma Corp., Seoul, South Korea). The lower and upper thirds of the right kidney were resected. After 1 week, the left kidney was removed. The rats of the sham group underwent the same incision and manipulation of the left and the right kidneys without tissue destruction. One week after the second surgical intervention, the rats in the Nx+STG group were fed a gelled diet containing 200 mg/kg/day of sitagliptin (MSD Korea, Seoul, South Korea), and the rats in the sham and Nx group were fed same gelled diet without sitagliptin. After 8 weeks of treatment, the animals were anesthetized with enflurane, blood samples were obtained, and the kidneys were collected. One portion of the right kidney was fixed in 10% phosphate-buffered formalin for morphologic and immunohistochemical analyses. The remainder of the right kidney was snap-frozen in liquid nitrogen and stored at −80°C for protein extraction.

### Physiologic measurements

Before and after the administration of a gelled diet with or without sitagliptin, the rats were weighed and placed in metabolic cages, and their urine was collected for 24 h. The urine volume was measured. Serum samples were taken from the tail vein. The blood glucose levels were measured by an Accu-check meter (Roche diagnostics, St Louis, MO, USA). BUN and creatinine levels in the serum and urine were measured using an automatic analyzer (ADVIA 1650, Siemens, Berlin, Germany). Creatinine clearance was calculated and adjusted for body weight.

### Determination of DPP IV enzymatic activity

DPP IV enzymatic activity was assayed in serum using DPP IV Activity Assay Kit (Biovision, Milpitas, CA, USA). A 50 μl volume of serum was diluted with 48 μl of DPP IV assay buffer and mixed with 2 μl substrate Gly-Pro-7-Amino-4-Methylcoumarin (AMC) and then incubated at 37°C for 30 min. The release of AMC from the substrate was measured with a fluorescence spectrophotometer at 360 nm of excitation and 460 nm of emission.

### Renal histologic and immunohistochemical analyses

Tissue for light microscopy and immunoperoxidase staining was fixed in formalin and embedded in paraffin. Three-micrometer sections were stained with hematoxylin and eosin (H&E). Apoptosis was detected with the enzymatic labeling of DNA strand breaks using terminal deoxynucleotidyl transferase-mediated deoxyuridine triphosphate nick end-labeling (TUNEL). TUNEL staining was performed with a Cell Death Detection kit (Roche, Mannheim, Germany). To reveal the total nuclei, the same slides were stained with 4’,6’-diamidino-2-phenyindole (DAPI) in phosphate-buffered saline. Indirect immunoperoxidase staining with an anti-ED-1 antibody (Serotec, Oxford, UK) was performed.

### Quantification of morphologic data

All analyses were performed in a blinded manner. Segmental and complete glomerular sclerosis was analyzed using a semiquantitative scoring system from 0 to 4 (0, no glomerulosclerosis; 1, <25% of glomerular area affected; 2, 25–50% affected; 3, 50–75% affected; and 4, 75–100% affected). At least 30 glomeruli were evaluated under × 400 magnification, and the results were averaged. The tubulointerstitial injury score was estimated based on the number of tubule dilatations, the distortion of the tubular basement membranes, and atrophy from 0 to 3 [0, none (<5%); 1, mild (5–25%); 2, moderate (25–50%); and 3, severe (>50%)]. More than 10 consecutive fields were examined under × 200 magnification, and the results were averaged. TUNEL (+) apoptotic nuclei were counted in more than 20 consecutive fields under × 200 magnification, and the results were averaged. The mean numbers of infiltrating macrophages (ED-1 positive cells) were calculated by averaging the total numbers of positive cells in more than 20 sequentially-selected, 0.25-mm^2^ grids at × 200 magnification.

### Western blot analysis

Whole kidneys were homogenized in lysis buffer (250 mM sucrose, 10 mM triethanolamine, 1 μg/ml leupeptin, and 0.1 mg/ml PMSF titrated to pH 7.6). The total protein concentration was measured using a bicinchoninic acid protein assay reagent kit (Sigma, St. Louis, MO, USA). The samples were run on SDS-polyacrylamide minigels (Bio-Rad Mini Protean III). The proteins were transferred to nitrocellulose membranes by electroelution. The following proteins were detected using specific antibodies: GLP-1R (Abcam, Cambridge, UK), β-actin (Santa Cruz Biotech, Santa Cruz, CA, USA), PI3K (BD Bioscience, Franklin Lakes, NJ, USA), total Akt, phospho-Ser473 Akt, total FoxO3a, phospho-Ser253 FoxO3a, total c-Jun N-terminal kinase (JNK), phospho-Thr183/Tyr185 JNK, caspase-3 (all from Cell Signaling Technology, Danvers, MA, USA), caspase-9 (BD Bioscience), Bax (Santa Cruz Biotech), and catalase (Abcam). After incubation with peroxidase-conjugated secondary antibodies (Pierce no. 31458, Rockford, IL, USA), bands were visualized using an enhanced chemiluminescence substrate (ECLTM RPN 2106; Amersham Pharmacia Biotech, Buckinghamshire, UK) before exposure to X-ray film (Hyperfilm; Amersham Pharmacia Biotech, Buckinghamshire, UK). The band densities were quantified by densitometry (GS-700 Imaging Densitometry, Bio-Rad, Hercules, CA, USA).

### Statistical analysis

All of the data are presented as the means ± S.D. The statistical analyses were performed using SPSS (version 15.0. for Windows; SPSS Inc., Chicago, IL, USA). Difference among three groups were tested with one-way analysis of variance (ANOVA), followed by Turkey’s multiple comparison post-test. Statistical significance was indicated by *P* < 0.05.

## Results

**Figure 1:**
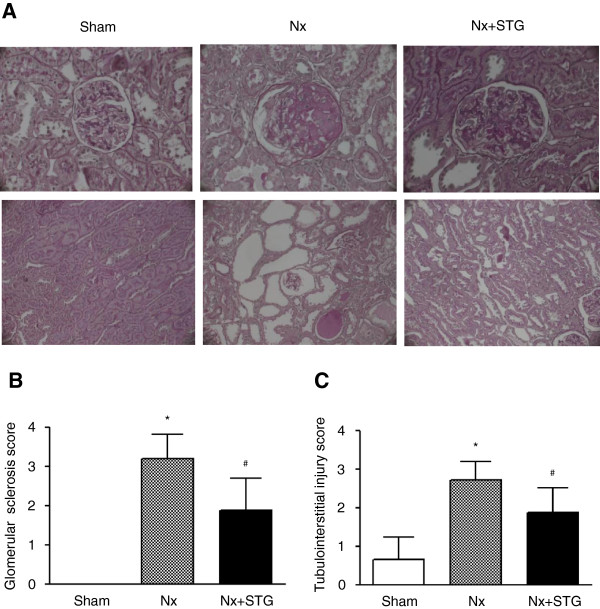
**Effects of sitagliptin on renal histology.** Representative images of H&E-stained renal tissues (**A**). Histological evaluation of glomerular sclerosis (**B**) and tubulointerstitial injury (**C**). The extent of glomerular sclerosis and tubulointerstitial injury was evaluated and scored. The results are expressed as the mean ± s.d. *Significantly different with respect to the sham-operated rats; ^#^significantly different with respect to the nephrectomized rats; *^#^*P* < 0.05. Sham, sham operation; Nx, nephrectomy; Nx+STG, nephrectomy and sitagliptin treatment. Magnification × 400 (upper panel) and x 200 (lower panel).

Table 1 shows the initial and final physiologic parameters of the animals. At the end of the study, rats that received a nephrectomy had a reduced body weight than the sham-operated rats did. However, sitagliptin treatment did not affect body weight or blood glucose levels in the animals. The BUN level remained increased after nephrectomy but was not significantly influenced by treatment with sitagliptin. Serum creatinine levels were increased in the nephrectomized rats, but they were significantly decreased by sitagliptin treatment. Sitagliptin also significantly improved creatinine clearance in nephrectomized rats. Figure 1 shows a representative histological image of the kidneys from each group of animals. In the nephrectomized rats that received sitagliptin, the severity of glomerulosclerosis was significantly reduced compared with the nephrectomized rats that were untreated (score: 1.88 ± 0.83 vs. 3.20 ± 0.63, *P* < 0.05). The degree of tubulointerstitial injury was also significantly reduced by sitagliptin in the nephrectomized rats (score: 1.88 ± 0.64 vs. 2.71 ± 0.49, *P* < 0.05). From these results, sitagliptin attenuated renal dysfunction and improved histological damage induced by subtotal nephrectomy.

**Table 1:** Physiologic parameters of experimental animals

	**Sham (n=6)**	**Nx (n=8)**	**Nx+STG (n=7)**
Body weight (g)			
Initial	205 ± 8	204 ± 9	199 ± 10
Final	563 ± 34	493 ± 29*	475 ± 31*
Blood glucose (mg/dl)			
Initial	111 ± 11	100 ± 10	111 ± 6
Final	180 ± 9	184 ± 21	172 ± 21
BUN (mg/dl)			
Initial	8.27 ± 2.48	8.63 ± 1.56	9.94 ± 3.41
Final	12.17 ± 2.50	28.19 ± 3.56*	25.04 ± 7.12*
Cr (mg/dl)			
Initial	0.39 ± 0.08	0.42 ± 0.06	0.39 ± 0.11
Final	0.52 ± 0.04	0.90 ± 0.09*	0.70 ± 0.19*^#^
Cl_Cr_ (ml/min/100 g)			
Initial	0.62 ± 0.25	0.52 ± 0.09	0.78 ± 0.33
Final	0.48 ± 0.05	0.31 ± 0.03*	0.43 ± 0.12^#^

The results are expressed as the mean ± s.d.

*Significantly different with respect to the sham-operated rats; ^#^significantly different with respect to the nephrectomized rats; *^#^*P* < 0.05.

Sham, sham operation; Nx, nephrectomy; Nx+STG, nephrectomy and sitagliptin treatment.

Cr, creatinine; Cl_Cr_, creatinine clearance normalized to body weight.

**Figure 2:**
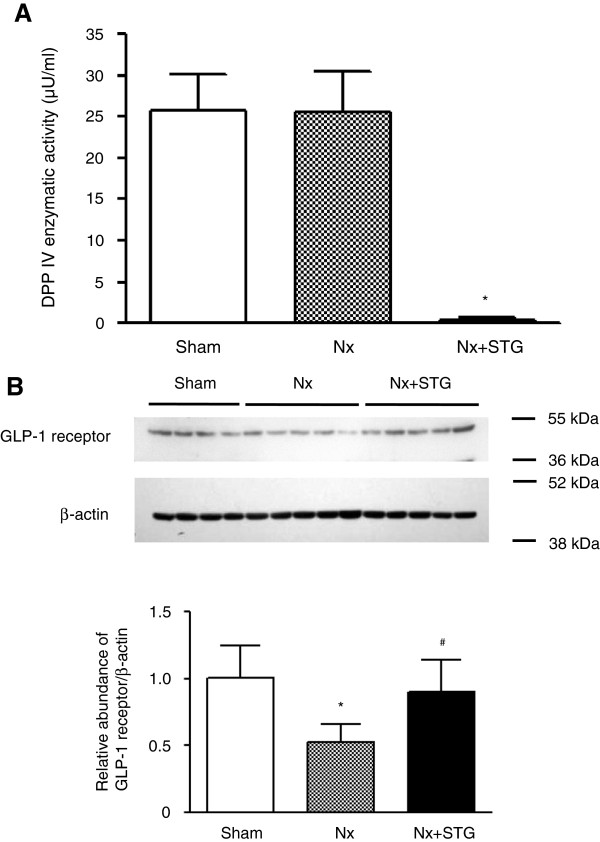
**Effects of sitagliptin on DPP IV activity and GLP-1 receptor expression.** DPP IV enzymatic activity in serum of rats from each group (**A**). Representative western blot and group data showing GLP-1 receptor protein abundance in the kidneys of rats from each group (**B**). The intensity of the bands corresponding to the GLP-1 receptor was corrected by β-actin levels to obtain relative measures of GLP-1 receptor expression among all samples. The results are expressed as the mean ± s.d. *Significantly different with respect to the sham-operated rats; ^#^significantly different with respect to the nephrectomized rats; *^#^*P* < 0.05. Sham, sham operation; Nx, nephrectomy; Nx+STG, nephrectomy and sitagliptin treatment.

There was no difference in DPP IV activity in the nephrectomized rats compared with the sham-operated rats (Figure 2A). An almost complete DPP IV inhibition was observed in serum of sitagliptin-treated rats with less than 2% residual DPP IV activity. The expression of GLP-1R in the kidney was reduced by half after 8 weeks of subtotal nephrectomy (Figure 2B). However, treatment with sitagliptin restored the expression of GLP-1R to the level of sham-operated rats.

**Figure 3:**
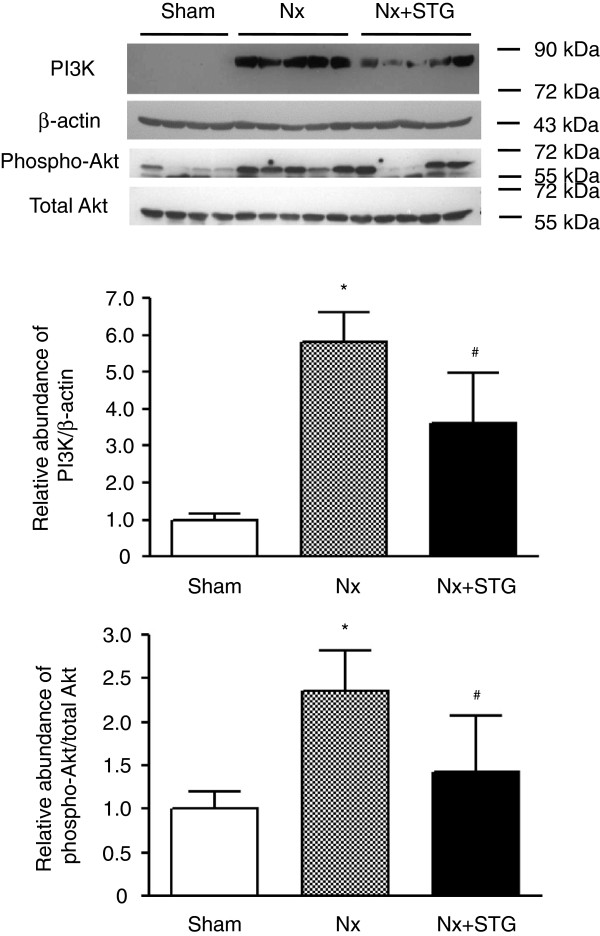
**Effects of sitagliptin on PI3K and Akt activity.** Representative western blot and group data showing PI3K protein abundance and Akt phosphorylation in the kidneys of rats from each group. The intensity of the bands corresponding to PI3K was corrected by β-actin levels, and phospho-Akt intensity was corrected by total Akt levels. The results are expressed as the mean ± s.d. *Significantly different with respect to the sham-operated rats; ^#^significantly different with respect to the nephrectomized rats; *^#^*P* < 0.05. Sham, sham operation; Nx, nephrectomy; Nx+STG, nephrectomy and sitagliptin treatment.

**Figure 4:**
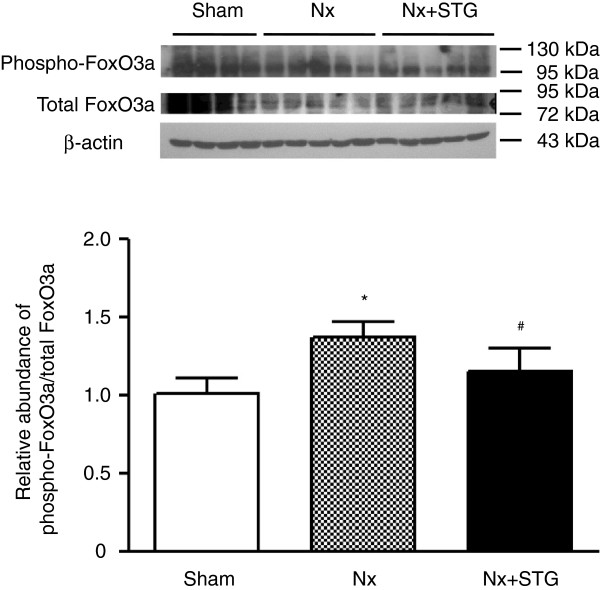
**Effects of sitagliptin on FoxO3a phosphorylation.** Representative western blot and group data showing FoxO3a phosphorylation in the kidneys of rats from each group. The intensity of the bands corresponding to phospho-FoxO3a was corrected by total FoxO3a levels to obtain relative measures of FoxO3a phosphorylation among all samples. The results are expressed as the mean ± s.d. *Significantly different with respect to the sham-operated rats; ^#^significantly different with respect to the nephrectomized rats; *^#^*P* < 0.05. Sham, sham operation; Nx, nephrectomy; Nx+STG, nephrectomy and sitagliptin treatment.

We determined the levels of PI3K and phospho-Akt in the kidneys. The levels of PI3K and phospho-Akt in the nephrectomized rats were significantly increased compared to the sham-operated rats, whereas sitagliptin treatment of nephrectomized rats significantly reduced both of these levels (Figure 3). Because PI3K activation and Akt phosphorylation serve as negative regulators of FoxO transcription factors, we next examined the phosphorylation of FoxO3a. Western blotting showed that the phospho-FoxO3a/total FoxO3a ratios were significantly increased in the nephrectomized rats compared to the sham-operated rats (Figure 4). However, the phospho-FoxO3a/total FoxO3a ratios were significantly decreased by sitagliptin treatment. Therefore, sitagliptin restored the inactivation of FoxO3a induced by subtotal nephrectomy.

**Figure 5:**
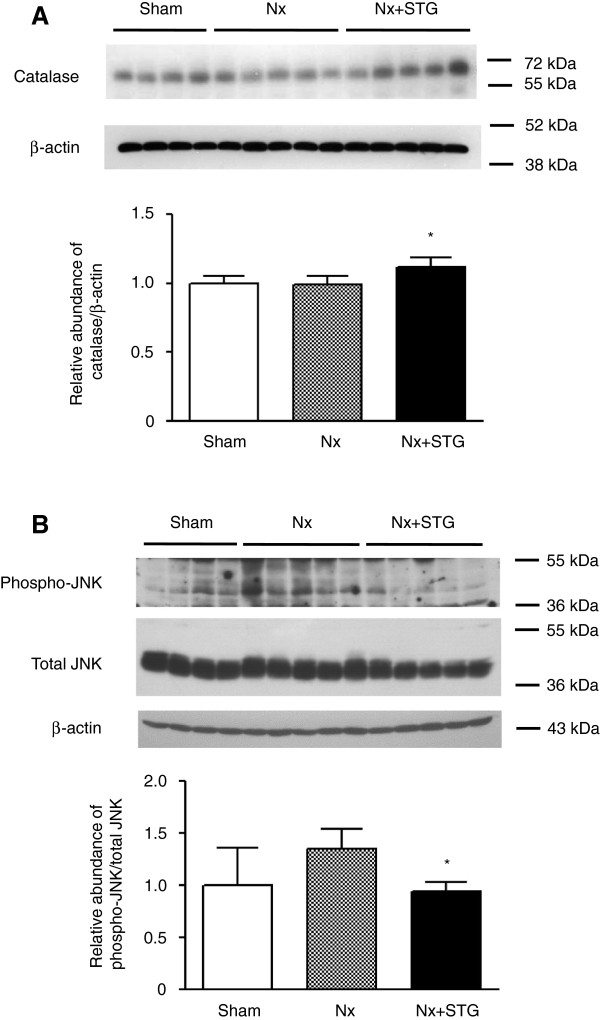
**Effects of sitagliptin on oxidative stress.** Representative western blot and group data showing catalase expression (**A**) and JNK phosphorylation (**B**) in the kidneys of rats from each group. The intensity of the bands corresponding to catalase was corrected by β-actin levels, and phospho-JNK intensity was corrected by total JNK levels. The results are expressed as the mean ± s.d. *Significantly different with respect to the nephrectomized rats; *P < 0.05. Sham, sham operation; Nx, nephrectomy; Nx+STG, nephrectomy and sitagliptin treatment; JNK, c-Jun N-terminal kinase.

To investigate whether the status of FoxO3a phosphorylation affected downstream signaling activity, we examined changes in the antioxidant protein catalase. As shown in Figure 5A, the expression of catalase was significantly increased by sitagliptin treatment. Because JNK is activated by oxidative stress, we next examined JNK phosphorylation. However, there was no difference of the phospho-JNK/total JNK ratios in the nephrectomized rats compared with the sham-operated rats (Figure 5B). The phospho-JNK/total JNK ratios were significantly decreased by sitagliptin treatment. From these results, the antioxidant effect of catalase decreased the activity of JNK in the nephrectomized rats after sitagliptin treatment.

**Figure 6:**
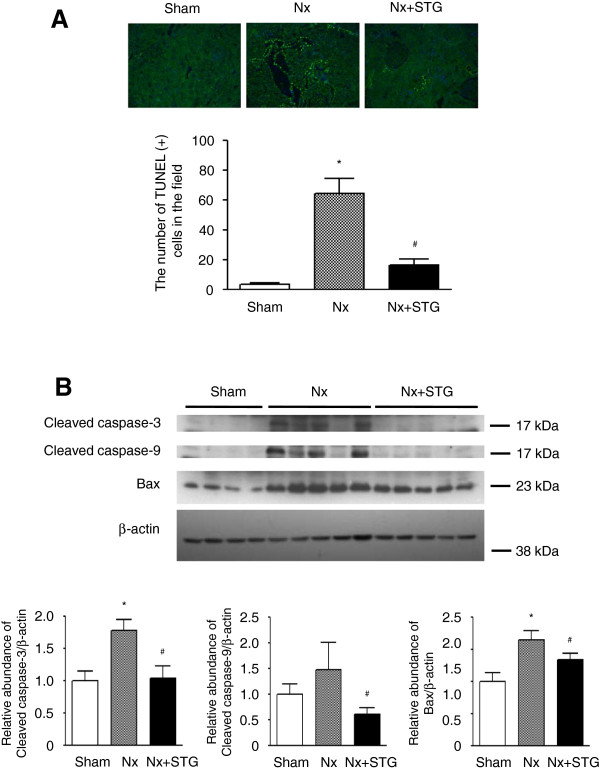
**Effects of sitagliptin on apoptosis.** Apoptotic cells detected in the kidneys stained with TUNEL (**A**). Representative western blot and group data showing cleaved caspase-3, cleaved caspase-9, and Bax protein abundance in the kidneys of rats from each group (**B**). The intensity of the bands corresponding to cleaved caspase-3, cleaved caspase-9, and Bax was corrected by β-actin levels. The results are expressed as the mean ± s.d. *Significantly different with respect to the sham-operated rats; ^#^significantly different with respect to the nephrectomized rats; *^#^*P* < 0.05. Sham, sham operation; Nx, nephrectomy; Nx+STG, nephrectomy and sitagliptin treatment. Magnification × 200.

To investigate the extent of apoptosis, we examined kidney sections after detecting DNA fragmentation with an *in situ* TUNEL assay. Scattered and bright nuclei stained by the TUNEL assay were easily detected in the kidneys of nephrectomized rats, but the number of nuclei was significantly decreased in the kidneys of the sitagliptin-treated rats (Figure 6A). Next, we examined changes in the proapoptotic proteins caspase-3, caspase-9, and Bax by western blot analysis. The cleaved subtypes of both caspase-3 and caspase-9, and Bax were increased in the kidneys of nephrectomized rats (Figure 6B). However, treatment with sitagliptin significantly reduced the levels of Bax and cleaved subtypes of both caspase-3 and caspase-9 in the nephrectomized rats. These results indicate that sitagliptin reduces the extent of apoptosis in the kidneys of nephrectomized rats.

**Figure 7:**
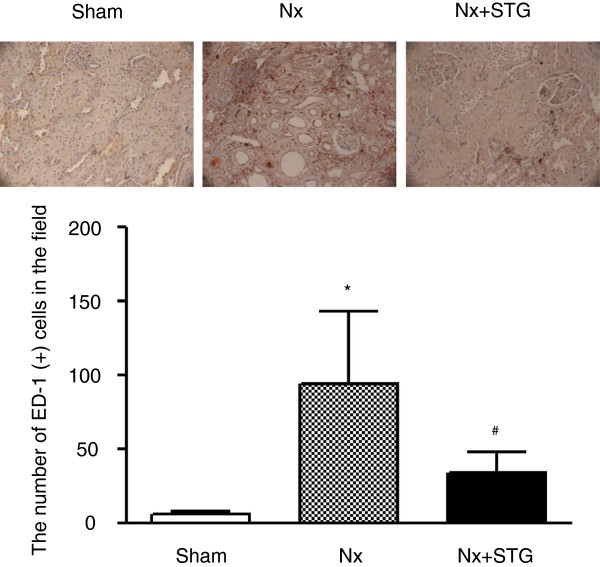
**Effects of sitagliptin on the infiltration of macrophages.** Immunohistochemical stainings for ED-1 in representative kidneys of experimental animals. The number of ED-1-positive cells per field was counted in each group. The results are expressed as the mean ± s.d. *Significantly different with respect to the sham-operated rats; ^#^significantly different with respect to the nephrectomized rats; *^#^*P* < 0.05. Sham, sham operation; Nx, nephrectomy; Nx+STG, nephrectomy and sitagliptin treatment. Magnification × 200.

Subtotal nephrectomy was associated with macrophage infiltration in the tubulointerstitium, as determined by an increase in ED-1-positive cells (Figure 7). After counting the absolute number of ED-1-positive cells, we observed a marked increase in macrophage infiltration after nephrectomy and a significant reduction in response to sitagliptin treatment. The mean ED-1 score was 94.29 ± 48.51 in nephrectomized rats and 34.33 ± 14.12 in sitagliptin-treated nephrectomized rats.

## Discussion

This study demonstrated that sitagliptin treatment after renal mass reduction showed a renoprotective effect. To the best of our knowledge, this report is the first one to show the effects of sitagliptin, a DPP IV inhibitor, on renal damage in the remnant kidney model. Sitagliptin ameliorated renal dysfunction and attenuated glomerular and tubulointerstitial injury in this model. Treatment with sitagliptin was found to exert anti-oxidative, anti-apoptotic, and anti-inflammatory effects in this model, together with the inactivation of the PI3K-Akt pathway and the resulting activation of FoxO3a.

In this study, sitagliptin, anti-diabetic drug, did not reduce blood glucose levels in the nephrectomized rats. Therefore, the renoprotective effect of sitagliptin is irrelevant to the reduction of glycemia. DPP IV inhibition does not cause hypoglycemia in a study in healthy male volunteers [19]. Because the action of GLP-1 on insulin secretion is strictly glucose dependent, the risk of hypoglycemia associated with DPP IV inhibitors is low [20].

In the kidney, GLP-1R is strongly expressed in both the glomeruli and proximal tubules [4,21]. However, it has been reported that its expression is reduced in diabetic kidneys [4,7]. In the heart, the GLP-1R mRNA expression was significantly reduced after subtotal nephrectomy [22]. We first found that GLP-1R expression was markedly decreased in the kidney after subtotal nephrectomy. Judging from the complete inhibition of DPP IV activity in sitagliptin-treated rats, we are sure that sitagliptin significantly raised plasma GLP-1 levels. It has been reported that GLP-1 agonist acts a renoprotective role through increasing GLP-1R expression in diabetic kidneys [4]. Therefore, chronic sitagliptin treatment in this study might activate renal GLP-1R through DPP IV inhibition because the protein abundance of GLP-1R was significantly increased in kidney homogenates. The dose of sitagliptin used in this study was far above the dose of anti-diabetic usage. To prove the tissue protective effects of DPP IV inhibition, we determined the dose from previous studies [13,23].

Currently, various target genes of FoxOs have been identified in insulin-responsive tissues [24,25]. Therefore, the connection between GLP-1 and FoxO has only been studied in pancreatic beta cells [26]. There are a few studies that have investigated FoxO signaling in the kidney [27,28]. Our study is the first to examine the association between GLP-1 and FoxO signaling in rat remnant kidneys. We only investigated the status of FoxO3 in this study because it is the most abundant protein among FoxO subfamily members. The activation of PI3K, the phosphorylation of Akt, and the inactivation of FoxO3a were the main pathway in this disease model. Sitagliptin treatment reversed this pathway.

Although the precise mechanism remains to be elucidated, CKD has been to known to be associated with oxidative stress [29,30]. Oxidative stress can occur either as a result of an increased ROS generation, a depressed antioxidant system or both. Catalase is a peroxidase enzyme that is of the major antioxidant defense systems [31]. However, catalase expression and JNK phosphorylation were not changed in this study. Future studies are needed to address these issues. GLP-1R activation using a GLP-1 analog or DPP IV inhibitor reduced oxidative stress in diabetic nephropathy and renal IRI [4,5,7,8]. The specific mechanism underlying the anti-oxidative effect of GLP-1R activation remains unclear. In this study, we speculate that the underlying mechanism might be the up-regulation of antioxidant catalase by FoxO3a activation through sitagliptin treatment.

An anti-apoptotic effect mediated by GLP-1R has been suggested in various tissues, including pancreatic beta cells [32], neurons [33], and cardiomyocytes [12]. GLP-1R activation also inhibited apoptosis in diabetic retinopathy [34] and diabetic nephropathy [4,7]. The underlying anti-apoptotic mechanism of GLP-1R has been reported in many *in vitro* studies [35-40]. GLP-1 is capable of inducing downregulation of the pro-apoptotic protein Bax, upregulation of the anti-apoptotic protein Bcl-2, phosphorylation and inactivation of Bad, reducing caspase-3 activity and DNA fragmentation.

Inflammatory cell infiltration induced by subtotal nephrectomy was attenuated by sitagliptin treatment in this study. A GLP-1R agonist showed anti-inflammatory effects in diabetic nephropathy [4,5]. In kidney IRI, GLP-1R activation using a DPP IV inhibitor ameliorated inflammation [8]. The anti-inflammatory effect of GLP-1R activation was also reported in the animal model of atherosclerosis [41]. Therefore, we speculate that GLP-1R activation by sitagliptin in a CKD animal model showed similar results.

Our study has some limitations. First, we performed the experiments with only three groups of animals without a group of animals with sham-operation and sitagliptin treatment. Due to treatment with a high dose of sitagliptin, we should have included this experimental group to observe any adverse effects in the animals. However, higher doses of sitagliptin than those used in our experiment have been proven to be safe in previous studies [12,18]. Moreover, our experiment showed no significant effects on body weight gain or the changes in blood glucose levels in the animals. Second, there is insufficient evidence that the beneficial effect of sitagliptin is through the activation of GLP-1R. DPP IV acts on a wide range of substrates. There is a possibility that other target molecules of DPP IV except GLP-1 may exert the renoprotective effects because plasma GLP-1 levels were not measured in this study. Knockout experiments inhibiting GLP-1 or GLP-1R would be required in the future. Third, there is no direct evidence to determine the causal relationship between GLP-1R and FoxO3a signaling. *In vitro* experiments using renal cells would also be required to study the direct effects of the GLP-1R on the signaling proteins (PI3K, Akt, JNK, and FoxO3a).

## Conclusions

In summary, sitagliptin treatment attenuated renal dysfunction and structural damage in a model of renal mass reduction. A reduction of apoptosis, inflammation and an increase of antioxidant could be suggested as a renoprotective mechanism, together with the activation of FoxO3a signaling. Therefore, DPP IV inhibitors might provide a promising approach for treating CKD, but their application in clinical practice remains to be investigated.

## Competing interests

The authors declare that they have no competing interests.

## Authors’ contributions

KWJ, JSH, KYN: participated in research design. KWJ, SK, JL: conducted experiments. SA, HJC, DWC: performed data analysis. KYN: wrote or contributed to the writing of the manuscript. All authors read and approved the final manuscript.

## Pre-publication history

The pre-publication history for this paper can be accessed here:

http://www.biomedcentral.com/1471-2369/14/98/prepub
